# Impact of polymorphisms in genes orchestrating innate immune responses on replication kinetics of Torque teno virus after kidney transplantation

**DOI:** 10.3389/fgene.2022.1069890

**Published:** 2022-11-22

**Authors:** Natalia Redondo, Isabel Rodríguez-Goncer, Patricia Parra, Eliseo Albert, Estela Giménez, Tamara Ruiz-Merlo, Francisco López-Medrano, Rafael San Juan, Esther González, Ángel Sevillano, Amado Andrés, David Navarro, José María Aguado, Mario Fernández-Ruiz

**Affiliations:** ^1^ Unit of Infectious Diseases, Hospital Universitario ‘12 de Octubre’, Instituto de Investigación Sanitaria Hospital ‘12 de Octubre’ (imas12), Madrid, Spain; ^2^ Centro de Investigación Biomédica en Red de Enfermedades Infecciosas (CIBERINFEC), Instituto de Salud Carlos III, Madrid, Spain; ^3^ Department of Microbiology, Instituto de Investigación Sanitaria INCLIVA, Hospital Clínico Universitario, Valencia, Spain; ^4^ Department of Medicine, School of Medicine, Universidad Complutense, Madrid, Spain; ^5^ Department of Nephrology, Instituto de Investigación Sanitaria Hospital “12 de Octubre” (imas12), Hospital Universitario “12 de Octubre”, Madrid, Spain; ^6^ Department of Microbiology, School of Medicine, University of Valencia, Valencia, Spain

**Keywords:** single-nucleotide polymorphisms, Torque teno virus, TTV replication kinetics, kidney transplantation, SNP

## Abstract

**Background:** Torque teno virus (TTV) DNAemia has been proposed as a surrogate marker of immunosuppression after kidney transplantation (KT), under the assumption that the control of viral replication is mainly exerted by T-cell-mediated immunity. However, Tthe impact on post-transplant TTV kinetics of single genetic polymorphisms (SNPs) in genes orchestrating innate responses remains unknown. We aimed to characterize the potential association between 14 of these SNPs and TTV DNA levels in a single-center cohort of KT recipients.

**Methods:** Plasma TTV DNAemia was quantified by real-time PCR in 221 KT recipients before transplantation (baseline) and regularly through the first 12 post-transplant months. We performed genotyping of the following SNPs: *CTLA4* (rs5742909, rs231775), *TLR3* (rs3775291), *TLR9* (rs5743836, rs352139), *CD209* (rs735240, rs4804803), *IFNL3* (rs12979860, rs8099917), *TNF* (rs1800629), *IL10* (rs1878672, rs1800872), *IL12B* (rs3212227) and *IL17A* (rs2275913).

**Results:** The presence of the minor G allele of *CD209* (rs4804803) in the homozygous state was associated with undetectable TTV DNAemia at the pre-transplant assessment (adjusted odds ratio: 36.96; 95% confidence interval: 4.72–289.67; *p*-value = 0.001). After applying correction for multiple comparisons, no significant differences across SNP genotypes were observed for any of the variables of post-transplant TTV DNAemia analyzed (mean and peak values, areas under the curve during discrete periods, or absolute increments from baseline to day 15 and months 1, 3, 6 and 12 after transplantation).

**Conclusion:** The minor G allele of *CD209* (rs4804803) seems to exert a recessive protective effect against TTV infection in non-immunocompromised patients. However, no associations were observed between the SNPs analyzed and post-transplant kinetics of TTV DNAemia. These negative results would suggest that post-transplant TTV replication is mainly influenced by immunosuppressive therapy rather than by underlying genetic predisposition, reinforcing its clinical application as a biomarker of adaptive immunity.

## Introduction

The study of the human virome in health and disease has gained growing attention over recent years (Webb et al., 2020; Dodi et al., 2021). Viruses belonging to *Anelloviridae* famil*y* are the most abundant eukaryotic viruses in the virome and may be detected in a variety of samples, such as blood, plasma, urine or saliva (Kaczorowska and van der Hoek, 2020; Arze et al., 2021). Anelloviruses are non-enveloped viruses with small circular replication-associated protein-encoding single-stranded DNA genomes (Biagini, 2009; Kaczorowska and van der Hoek, 2020), which lack attributable pathogenic roles (“orphan viruses”) (Focosi et al., 2016; Rezahosseini et al., 2019). Once primary infection occurs at early stages of life, anelloviruses remain in different body compartments and fluids—including peripheral blood mononuclear cells, feces, semen, throat swabs, umbilical cord blood, lungs, kidneys or cerebrospinal fluid—under the control of the immune system, resulting in a prevalence as high as 90% in the adult population (Redondo et al., 2022a). The precise underlying mechanisms on how this immune control is carry out largely remain to be determined, although a major role has been proposed for the cellular arm. Belonging to the *Alphatorquevirus* genus and discovered in 1997 (Nishizawa et al., 1997), Torque teno virus (TTV) has been proven by us and others to serve as a convenient surrogate marker of the overall status of immunosuppression after solid organ (SOT) and allogeneic hematopoietic stem cell transplantation (HSCT) (Fernandez-Ruiz et al., 2019; Rezahosseini et al., 2019; Mouton et al., 2020; Redondo et al., 2022a; Jaksch et al., 2022).

The innate immunity acts as a frontline defense against viruses through an orchestrated response, that is, triggered upon recognition of viral motifs by pathogen recognition receptors (PRRs) present in macrophages and dendritic cells (Takeuchi and Akira, 2010). The rationale for the use of TTV DNAemia as a biomarker of immune competence after SOT lies on the assumption that the viral kinetics is mainly dictated by the T-cell-mediated immunity (Redondo et al., 2022a; Jaksch et al., 2022). Indeed, various studies have shown a direct correlation between TTV DNA loads and calcineurin inhibitors trough levels (Gorzer et al., 2014; Jaksch et al., 2018). The role played by the innate immune arm in the setting of ongoing immunosuppression remains largely unknown, as is the potential impact of polymorphisms in genes coding for PRRs (such as toll-like receptors [TLRs]), interleukins (IL) or interferons (IFNs) (Prasetyo et al., 2015; Ramzi et al., 2019; Ramzi et al., 2021). Evidence of an individual genetic susceptibility to TTV regardless of the amount of immunosuppressive therapy would question the reliability of viral replication as clinical biomarker in the SOT population.

We aimed to investigate the association between 14 single genetic polymorphisms (SNPs) in different genes mainly involved in the orchestration of innate immune responses (Table 1) and TTV DNA levels at baseline and various points during the first post-transplant year in a well characterized cohort of kidney transplant (KT) recipients (Fernandez-Ruiz et al., 2019). The selection of these SNPs was dictated by previous research showing a potential impact on the susceptibility to viral infections. In the case of *TLR3* (rs3775291), various pieces of evidence have shown an effect on the incidence of infection by cytomegalovirus (CMV) or BK polyomavirus (BKPyV), two relevant viral pathogens in the KT scenario, but also tick-borne encephalitis, chikungunya or hepatitis B virus (HBV) (Kindberg et al., 2011; Geng et al., 2016; Studzinska et al., 2017; Fischer et al., 2018; Bucardo et al., 2021; Redondo et al., 2022b). We have previously reported that certain SNPs in *TLR9* (rs5743836, rs352139) modulate the risk of CMV infection in two independent cohorts of KT recipients (Fernandez-Ruiz et al., 2015; Redondo et al., 2022b). Regarding SNPs located in the *CD209* gene, rs735240 appears to increase the incidence of CMV infection in seropositive KT recipients not receiving antiviral prophylaxis (Fernandez-Ruiz et al., 2015), whereas rs4804803 has been correlated with an increased susceptibility to dengue virus (Vargas-Castillo et al., 2018) and, more recently, BKPyV (Redondo et al., 2022c). We analyzed the SNPs located in *IFNL4* (rs12979860, rs8099917) due to its well-established relevance in other viral infections, including CMV (Fernandez-Ruiz et al., 2015) and hepatitis C virus (HCV) (Ge et al., 2009; Thomas et al., 2009). Finally, we aimed to validate in the SOT population the associations reported by other authors between *CTLA4* (rs5742909, rs231775), *TNF* (rs1800629), *IL10* (rs1800872, rs1878672), *IL12B* (rs3212227) and *IL17* (rs2275913) SNPs and the kinetics of TTV DNAemia after HSCT (Ramzi et al., 2019; Ramzi et al., 2021).

**TABLE 1:** Candidate SNPs selected for the present study.

Gene	Encoded protein	Biological function	SNP ID number	Nucleotide substitution (reference allele/ alternative allele)	Global allele frequency^a^	Impact of the SNP on the susceptibility to infection in previous studies^b^
*CTLA4*	Cytotoxic T-lymphocyte antigen 4 (CTLA-4/CD152)	T-cell co-inhibitory receptor	rs5742909	C / T	C = 0.91755	Increased risk of CMV after SOT (Misra et al. 2015)
					T = 0.08245	
			rs231775	A / G	A = 0.628256	Increased risk of chronic HCV in the general population (Ali et al. 2022) and CMV after SOT (Misra et al. 2015)
					G = 0.371744	
*TLR3*	Toll-like receptor 3: endosomal PRR	Endocytic pathogen recognition receptor of single and double-stranded RNA	rs3775291	C / T	C = 0.716526	Increased risk of CMV (Redondo et al. 2022b) and BKPyV after SOT (Redondo et al. 2022c), increased risk of dengue (Singh et al. 2021) and HBV in the general population (Ye et al. 2020)
					T = 0.283474	
*TLR9*	Toll-like receptor 9: endosomal PRR	Recognition of unmethylated CpG motif-containing DNA	rs5743836	A / G	A = 0.80444	Protection against TB (Varshney et al. 2022), increased risk of dengue (Singh et al. 2021), higher HBV viral load (Chihab et al. 2019) in the general population
					G = 0.19556	
			rs352139	T / C	T = 0.458978	Increased risk of CMV after SOT (Redondo et al. 2022b), increased risk of EBV-related IM in the general population (Jablonska et al. 2020)
					C = 0.541022	
*CD209*	Dendritic cell-specific ICAM 3-grabbing nonintegrin (DC-SIGN/CD209): endosomal C-type lectin receptor	Recognition of carbohydrates present in viruses, bacteria, fungi and parasites and DAMPs in damaged host T-cells	rs735240	G / A	G = 0.57414	Increased risk of CMV after SOT (Fernandez-Ruiz et al. 2015) and HSCT (Mezger et al. 2008)
					A = 0.42586	
			rs4804803	A / G	A = 0.786719	Protection against BKPyV after SOT (Redondo et al. 2022c), protection against severe dengue (Sakuntabhai et al. 2005) and TBE (Czupryna et al. 2017) and increased risk of symptomatic CHIKV (Chaaithanya et al. 2016) in the general population
					G = 0.213281	
*IFNL4*	Interferon-λ3 (IL28B), type III interferon: soluble immune mediator	Antiviral cytokine	rs12979860	C / T	C = 0.672446	Lower HCV clearance upon IFN-α therapy in the general population (Miri et al. 2021), protection against CMV after SOT (Fernandez-Ruiz et al. (2015) and HSCT (Bravo et al. 2014)
					T = 0.327554	
			rs8099917	T / G	T = 0.808472	Lower HCV clearance upon IFN-α therapy in the general population (Li et al. 2016), protection against CMV after SOT (Egli et al. 2014)
					G = 0.191528	
*TNF*	Tumor necrosis factor	Pro-inflammatory cytokine	rs1800629	G / A	G = 0.847933	Increased risk of severe influenza (Alagarasu et al. 2021) and COVID-19 (Gupta et al. 2022) in the general population
					A = 0.152067	
*IL10*	Interleukin-10: human cytokine	Pleiotropic cytokine	rs1800872	T / G	T = 0.29385	Increased risk of BKPyV after SOT (Redondo et al. 2022c)
					G = 0.70615	
			rs1878672	G / C	G = 0.68890	No apparent impact on the risk of CNV after HSCT (Corrales et al. 2015)
					C = 0.31110	
*IL12B*	Interleukin-12: human cytokine	Pro-inflammatory cytokine, T-cell and NK proliferation	rs3212227	T / G	T = 0.784650	Higher CMV viruria in newborns with congenital infection (Jedlinska-Pijanowska et al. 2021)
					G = 0.215350	
*IL17*	Interleukin-17: human cytokine	Pro-inflammatory cytokine, cell trafficking, immune modulation, induction of innate immunity, tissue repair	rs2275913	G / A	G = 0.665743	Increased risk of cutaneous leishmaniasis (Goncalves de Albuquerque et al. 2019) and protection against TB (Eskandari-Nasab et al. 2018) in the general population
					A = 0.334257	

^a^ Obtained from ALFA Allele Frequency (available at: https://www.ncbi.nlm.nih.gov/snp/).

^b^ The clinical effect associated with the minor (alternative) allele of the corresponding SNP is detailed.

BKPyV, BK polyomavirus; CHIK, chikungunya virus; CMV, cytomegalovirus; COVID-19, coronavirus disease 2019; CTLA-4, cytotoxic T-lymphocyte antigen 4; DAMP, damage-associated molecular pattern; HBV, hepatitis B virus; HCV, hepatitis C virus; HSCT, hematopoietic stem cell transplantation; IFN, interferon; IL, interleukin; IM, infectious mononucleosis; NK, natural killer; PRR, pattern recognition receptor; SD, standard deviation; SNP, single-nucleotide polymorphism; SOT, solid organ transplantation; TB, tuberculosis; TLR, toll-like receptor; TNF, tumor necrosis factor; TTV, torque teno virus.

## Material and methods

### Study population and setting

The present research was performed as a *post hoc* retrospective analysis of a previous study that investigated the role of TTV DNA levels to predict the occurrence of serious and opportunistic infection and *de novo* malignancy in a cohort of KT recipients recruited at the University Hospital “12 de Octubre” (a 1,300-bed tertiary care center in Madrid with an active KT program since 1990) between November 2014 and December 2016 (Fernandez-Ruiz et al., 2019). As detailed elsewhere, adult patients with end-stage renal disease (ESRD) undergoing KT during the study period and providing informed consent were eligible for inclusion. Exclusion criteria included double organ transplantation and primary graft non-function. By applying these criteria, 221 KT recipients were eventually included. The study was performed in accordance with the ethical standards laid down in the Declarations of Helsinki and Istanbul. The local Clinical Research Ethics Committee approved the study protocol.

### Study design

Participants were enrolled at the time of KT and followed-up for at least 12 months, unless graft loss (retransplantation or return to dialysis) or death occurred earlier. Plasma TTV DNA load was quantified at baseline (i.e., within 6 h prior to the transplant procedure), day 7, and months 1, 3, 6 and 12 by a polymerase chain reaction (PCR)-based quantitative nucleic acid amplification test. Immunosuppression and prophylaxis regimens are detailed as Supplementary Material.

### Single genetic polymorphisms genotyping

Whole blood specimens that have been stored at −70°C were retrieved for SNP genotyping. DNA was extracted with the KingFisher Duo Prime system using the MagMax DNA Multi-Sample Ultra 2.0 kit (Thermo Fisher Scientific, Waltham, MA) following the manufacturer’s instructions. *CTLA4* (rs5742909, rs231775), *TLR3* (rs3775291), *TLR9* (rs5743836, rs352139), *CD209* (rs735240, rs4804803), *IFNL3* (rs12979860, rs8099917), *TNF* (rs1800629), *IL10* (rs1878672, rs1800872), *IL12B* (rs3212227) and *IL17A* (rs2275913) genotyping was performed by Taqman technology (Thermo Fisher Scientific) in a QuantStudio 3 real-time PCR system (Applied Biosystems, Foster City, CA). SNP and allele calling was made by means of the TaqMan Genotyper Software version 1.0 (Applied Biosystems) and the QuantStudio Design and Analysis Software version 1.5.1 (ThermoFisher Scientific).

### Plasma torque teno virus DNA load quantification

TTV DNA extraction and quantification was performed as previously described (Fernandez-Ruiz et al., 2019). Briefly, DNA was extracted from 200 μL of plasma with the NucliSENSR easyMAGR automated system (bioMérieux, Marcy‐l’Étoile, France), following the manufacturer’s instructions. DNA loads were quantified by means of a real‐time PCR assay targeting a highly conserved segment of the 5′untranslated region of the viral genome (TTV R-gene kit, ARGENE range, bioMérieux). PCR amplification and amplicon detection was performed on an ABI Prism 7500 system (PE Biosystems, Foster City, CA). The viral load (in copy numbers per mL) was determined using a standard curve with known copy numbers and log10‐transformed for statistical analyses. The lower limit of detection (LLoD) was 167 copies/mL [95% confidence interval (CI): 92‐581] or 2.2 log^10^ copies/mL (95% CI: 2.0–2.8), with DNA quantitation in the linear range from 2.1 × 10^2^ to 2.1 × 10^7^ copies/ml. Specimens with undetectable DNA loads were assigned a value of 0.01 (−2.0 log^10^) copies/mL for analysis purposes. All samples from each patient were simultaneously assayed in singlets.

### Statistical analysis

Quantitative data were reported as the mean ± standard deviation (SD) or the median with interquartile range (IQR). Qualitative variables were given as absolute and relative frequencies. Normality of the distributions was tested with the Kolgomorov-Smirnov test. Deviation from the Hardy-Weinberg equilibrium for each SNP was evaluated by the χ^2^ test with one degree of freedom. Comparisons of TTV kinetics at different points across SNP genotypes were performed by the χ^2^ test or the Fisher’s exact test for qualitative variables (i.e. detectable or undetectable [below the LLoD] DNAemia), or by the T-Student or U-Mann-Whitney tests for continuous variables (i.e. plasma DNA levels). In addition, other viral kinetic parameters were compared across SNPs: peak plasma TTV DNA levels and areas under the curve (AUCs) for TTV DNAemia through discrete time periods (1, 3, and 6 months after transplantation), and increments (Δ) in DNA levels from baseline to day 15 and months 1, 3, 6 and 12. Additional pairwise comparisons were conducted between different SNP genotype groups, either individually or in combination. The independent impact of selected SNPs on the probability of having undetectable TTV DNAemia was confirmed by logistic regression, with associations given as odds ratios (ORs) and 95% CIs. All the significance tests were two-tailed and considered as significant at a *p*-value < 0.05. To control for *p*-value inflation due to multiple comparisons, the Bonferroni method (corrected α value = nominal α value/total number of comparisons) was applied. Statistical analysis was performed using SPSS version 21 (Statistical Package for Social Sciences, Chicago, IL).

## Results

We included 221 KT recipients, whose demographics, clinical characteristics and patient and graft outcomes are detailed in Table 2. Samples from all the patients were successfully genotyped for the 14 SNPs considered. The median number of assessments for plasma TTV DNA per patient was 5 (IQR: 4–5). The majority of recipients had detectable TTV DNAemia (i.e. above the LLoD) at every time point, ranging from 96.3% (180/187) at baseline to 99.4% (176/177) at post-transplant month 6. The genotypic frequencies of candidate SNPs are shown in Supplementary Table S1. The observed genotype frequency distributions did not deviate from those expected according to the Hardy-Weinberg equilibrium except for *TLR9* (rs5743836) and *IFNL3* (rs12979860).

**TABLE 2:** Demographic and clinical characteristics of the study cohort (n = 221).

Variable	
Age of recipient, years [mean ± SD]	53.9 ± 15.7
Male gender of recipient [n (%)]	160 (72.4)
Current or prior smoking history [n (%)]	90 (40.7)
Pre-transplant chronic co-morbidities [n (%)]	
Hypertension	188 (85.1)
Diabetes mellitus	70 (31.7)
Chronic lung disease	29 (13.1)
Coronary heart disease	22 (10.0)
Other chronic heart disease	39 (17.6)
Peripheral arterial disease	21 (9.5)
Cerebrovascular disease	18 (8.1)
Type of transplant [n (%)]	
Single kidney	206 (93.2)
Double kidney	15 (6.8)
Previous solid organ transplantation [n (%)]	29 (13.1)
Underlying end-stage renal disease [n (%)]	
Diabetic nephropathy	45 (20.4)
Polycystic kidney disease	26 (11.8)
Glomerulonephritis	50 (22.6)
IgA nephropathy	25 (11.3)
Nephroangiosclerosis	20 (9.0)
Chronic interstitial nephropathy	12 (5.4)
Congenital nephropathy	10 (4.5)
Reflux nephropathy	6 (2.7)
Lupus nephropathy	4 (1.8)
Vasculitis	5 (2.3)
Amiloidosis	3 (1.4)
Unknown	25 (11.3)
Other	30 (13.6)
CMV serostatus [n (%)]	
D+/R+	157 (71.0)
D+/R-	28 (12.7)
D-/R+	24 (10.9)
D-/R-	8 (3.6)
D unknown/R+	4 (1.8)
Positive HCV serostatus [n (%)]	17 (7.7)
Positive HIV serostatus [n (%)]	2 (0.9)
Pre-transplant renal replacement therapy [n (%)]	
Hemodialysis	159 (71.9)
Continuous ambulatory peritoneal dialysis	35 (15.8)
Time on dialysis, months [median (IQR)]	17.6 (9.3 – 35.3)
Age of donor, years [mean ± SD]	52.5 ± 16.1
Male gender of donor [n (%)]	117 (52.9)
Type of donor [n (%)]	
DBD donor	144 (65.2)
DCD donor	47 (21.3)
Living donor	29 (13.1)
Cold ischemia time, hours [median (IQR)]	17.0 (9.0 – 22.0)
Number of HLA mismatches [median (IQR)]	4 (3–5)
Induction therapy [n (%)]	
ATG	106 (48.0)
Basiliximab	85 (38.5)
None	30 (13.6)
Immunosuppression regimen at discharge [n (%)]	
Prednisone, tacrolimus and MMF/MPS	219 (99.1)
Prednisone, tacrolimus and azathioprine	16 (7.2)
Conversion to mTOR during follow-up [n (%)]	22 (10.0)
Time to conversion, days [median (IQR)]	217 (117 – 306.8)
Anti-CMV prophylaxis [n (%)]	125 (56.6)
Duration of prophylaxis, days [median (IQR)]	103.5 (91 – 148.5)
Follow-up period, days [median (IQR)]	494 (434 – 542)
Post-transplant complications [n (%)]	
Delayed graft function	102 (46.2)
Number of dialysis sessions [median (IQR)]	2 (1–3)
Reintervention within the first month	24 (10.9)
New-onset diabetes	22 (10.0)
Renal artery stenosis	40 (19.6)
Acute graft rejection^a^	25 (11.3)
Time to the first episode, days [median (IQR]	111 (19 – 159)
T-cell-mediated acute rejection	13 (5.9)
Antibody-mediated acute rejection	6 (2.7)
Graft loss [n (%)]	5 (2.3)
Time from transplantation, days [median (IQR)]	41 (18 – 260.5)
All-cause mortality [n (%)]	2 (0.9)

^a^ Includes 16 patients with borderline acute rejection and 14 with empirically-treated episodes without histological confirmation.

ATG, antithymocyte globulin; CMV, cytomegalovirus; D, donor; DBD, donation after brain death; DCD, donation after circulatory death; HCV, hepatitis C virus; HIV, human immunodeficiency virus; HLA, human leukocyte antigen; IQR, interquartile range; MMF/MPS, mycophenolate mofetil/enteric-coated mycophenolate sodium; R, recipient; SD, standard deviation.

First, the effect of studied polymorphisms on plasma TTV DNAemia at discrete time points was investigated. In particular, we explored the impact of the minor alleles in each SNP in both dominant (heterozygous and homozygous) and recessive (homozygous only) models. Across the 14 SNPs considered, we did not find significant differences in TTV DNA levels at any of the monitoring points (Table 3).

**TABLE 3:** Plasma TTV DNA levels at different post-transplant time points according to candidate SNPs.

SNP (ID number)	Model		Plasma TTV DNA level, log_10_ copies/mL (mean ± SD)											
			Baseline	*p*-value	Day 15	*p*-value	Month 1	*p*-value	Month 3	*p*-value	Month 6	*p*-value	Month 12	*p*-value
*CTLA4* (rs5742909)	Dominant	CC	2.9 ± 1.6	0.284	3.2 ± 1.6	0.389	4.4 ± 1.7	0.521	5.9 ± 1.8	0.495	5.3 ± 2.4	0.255	4.6 ± 1.9	0.496
		CT/TT	2.6 ± 1.5		2.9 ± 1.9		4.2 ± 1.6		5.7 ± 1.5		5.0 ± 1.9		4.8 ± 1.8	
	Recessive	CC/CT	2.9 ± 1.6	0.463	3.1 ± 1.7	0.821	4.4 ± 1.7	0.441	5.9 ± 1.7	0.620	5.3 ± 2.3	0.702	4.6 ± 1.9	0.237
		TT	3.5 ± 1.0		3.3 ± 1.0		5.0 ± 1.2		6.3 ± 1.8		5.8 ± 1.8		5.6 ± 1.7	
*CTLA4* (rs231775)	Dominant	AA	2.8 ± 1.4	0.705	3.1 ± 1.6	0.972	4.5 ± 1.5	0.364	5.9 ± 1.7	0.961	5.0 ± 2.3	0.013	4.5 ± 1.9	0.502
		AG/GG	2.9 ± 1.8		3.1 ± 1.7		4.3 ± 1.9		5.9 ± 1.8		5.6 ± 2.3		4.7 ± 1.9	
	Recessive	AA/AG	2.9 ± 1.6	0.601	3.1 ± 1.7	0.689	4.5 ± 1.7	0.233	5.9 ± 1.7	0.527	5.2 ± 2.3	0.126	4.6 ± 4.6	0.345
		GG	3.1 ± 1.7		3.3 ± 1.2		4.0 ± 1.9		6.1 ± 2.0		5.8 ± 2.7		5.0 ± 1.3	
*TLR3* (rs3775291)	Dominant	CC	2.9 ± 1.3	0.703	3.1 ± 1.7	0.730	4.2 ± 1.6	0.147	6.1 ± 1.8	0.158	5.5 ± 2.4	0.506	4.8 ± 1.9	0.254
		CT/TT	2.8 ± 1.9		3.2 ± 1.6		4.6 ± 1.8		5.7 ± 1.6		5.1 ± 2.2		4.5 ± 1.9	
	Recessive	CC/CT	2.8 ± 1.6	0.500	3.1 ± 1.6	0.917	4.4 ± 1.7	0.923	5.9 ± 1.8	0.908	5.3 ± 2.3	0.406	4.6 ± 1.9	0.939
		TT	3.1 ± 2.0		3.1 ± 2.0		4.4 ± 2.0		5.8 ± 1.4		4.9 ± 2.2		4.6 ± 1.7	
*TLR9* (rs5743836)	Dominant	AA	2.9 ± 1.6	0.337	3.2 ± 1.6	0.692	4.4 ± 1.7	0.829	5.8 ± 1.8	0.566	2.5 ± 2.5	0.847	4.6 ± 1.9	0.960
		AG/GG	2.7 ± 1.8		3.1 ± 1.8		4.4 ± 1.6		6.0 ± 1.7		5.4 ± 1.9		4.6 ± 2.0	
	Recessive	AA/AG	2.9 ± 1.6	0.733	3.1 ± 1.7	0.932	4.4 ± 1.7	0.640	5.9 ± 1.7	0.116	5.3 ± 2.4	0.650	4.6 ± 1.9	0.775
		GG	2.7 ± 1.0		3.2 ± 1.5		4.2 ± 1.4		5.0 ± 1.4		5.1 ± 1.8		4.8 ± 2.1	
*TLR9* (rs352139)	Dominant	TT	3.0 ± 1.8	0.388	3.1 ± 2.0	0.935	4.3 ± 2.1	0.488	6.2 ± 1.9	0.132	5.1 ± 2.4	0.764	4.4 ± 1.8	0.350
		TC/CC	2.8 ± 1.6		3.1 ± 1.5		4.5 ± 1.5		5.8 ± 1.7		5.4 ± 2.3		4.7 ± 1.9	
	Recessive	TT/TC	2.9 ± 1.6	0.508	3.1 ± 1.6	0.521	4.3 ± 1.8	0.184	6.0 ± 1.8	0.111	5.3 ± 2.5	0.703	4.6 ± 1.9	0.813
		CC	2.7 ± 1.7		3.3 ± 1.7		4.7 ± 1.5		5.6 ± 1.6		5.3 ± 1.7		4.6 ± 2.0	
*CD209* (rs735240)	Dominant	GG	2.8 ± 1.8	0.560	3.0 ± 1.9	0.532	4.6 ± 1.8	0.376	5.9 ± 1.8	0.883	5.2 ± 2.6	0.994	4.6 ± 1.7	0.937
		GA/AA	2.9 ± 1.5		3.2 ± 1.5		4.3 ± 1.7		5.9 ± 1.7		5.3 ± 2.2		4.6 ± 2.0	
	Recessive	GG/GA	2.8 ± 1.6	0.163	3.1 ± 1.7	0.808	4.4 ± 1.8	0.486	5.9 ± 1.7	0.907	5.3 ± 2.4	0.424	4.6 ± 1.9	0.571
		AA	3.2 ± 1.7		3.2 ± 1.4		4.6 ± 1.4		5.9 ± 1.8		5.2 ± 2.1		4.7 ± 1.8	
*CD209* (rs4804803)	Dominant	AA	2.9 ± 1.6	0.760	3.1 ± 1.5	0.726	4.4 ± 1.6	0.724	5.9 ± 1.6	0.927	5.1 ± 2.2	0.043	4.6 ± 1.7	0.897
		AG/GG	2.8 ± 1.7		3.2 ± 1.8		4.4 ± 1.9		5.9 ± 1.9		5.6 ± 2.5		4.6 ± 2.1	
	Recessive	AA/AG	2.9 ± 1.5	0.294	3.2 ± 1.6	0.373	4.4 ± 1.7	0.924	5.9 ± 1.7	0.658	5.3 ± 2.3	0.801	4.6 ± 1.9	0.578
		GG	1.6 ± 3.2		2.5 ± 2.5		4.5 ± 2.6		6.1 ± 2.0		5.2 ± 2.8		4.3 ± 2.4	
*IFNL3* (rs12979860)	Dominant	CC	3.0 ± 1.7	0.279	3.3 ± 1.6	0.330	4.6 ± 1.6	0.090	5.9 ± 1.7	0.712	5.5 ± 2.3	0.080	4.7 ± 2.1	0.392
		CT/TT	2.7 ± 1.6		3.0 ± 1.7		4.2 ± 1.8		5.8 ± 1.8		5.1 ± 2.3		4.5 ± 1.7	
	Recessive	CC/CT	2.8 ± 1.7	0.296	3.1 ± 1.7	0.733	4.5 ± 1.7	0.267	5.6 ± 1.7	0.644	5.3 ± 2.4	0.408	4.6 ± 1.9	0.644
		TT	3.2 ± 1.3		3.2 ± 1.6		4.1 ± 1.9		6.0 ± 1.8		5.2 ± 2.2		4.8 ± 1.9	
*IFNL3* (rs8099917)	Dominant	TT	2.9 ± 1.5	0.496	3.2 ± 1.6	0.827	4.4 ± 1.8	0.564	5.9 ± 1.7	0.471	5.4 ± 2.5	0.180	4.6 ± 2.0	0.901
		TG/GG	2.7 ± 1.8		3.1 ± 1.8		4.3 ± 1.7		5.7 ± 1.7		5.1 ± 1.8		4.6 ± 1.5	
	Recessive	TT/TG	2.8 ± 1.6	0.398	3.1 ± 1.7	0.583	4.4 ± 1.7	0.913	5.8 ± 1.7	0.227	5.3 ± 2.3	0.610	4.6 ± 1.9	0.682
		GG	3.3 ± 1.5		3.4 ± 1.1		4.3 ± 1.1		6.6 ± 1.2		5.6 ± 1.6		4.9 ± 1.6	
*TNF* (rs1800629)	Dominant	GG	2.9 ± 1.5	0.296	3.2 ± 1.6	0.131	4.5 ± 1.7	0.096	5.9 ± 1.7	0.313	5.4 ± 2.3	0.294	4.8 ± 1.7	0.077
		GA/AA	2.7 ± 1.9		2.8 ± 1.7		4.0 ± 1.6		5.6 ± 2.0		4.9 ± 2.5		4.1 ± 2.5	
	Recessive	GG/GA	2.9 ± 1.6	0.523	3.1 ± 1.6	0.539	4.4 ± 1.7	0.484	5.9 ± 1.7	0.618	5.3 ± 2.3	0.898	4.6 ± 1.9	0.964
		AA	3.6 ± 1.6		3.7 ± 1.5		5.1 ± 0.4		6.4 ± 1.8		5.4 ± 0.8		4.6 ± 1.8	
*IL10* (rs1800872)	Dominant	TT	2.6 ± 2.1	0.539	2.9 ± 1.2	0.477	4.3 ± 1.5	0.858	5.8 ± 1.4	0.807	5.1 ± 2.3	0.970	4.8 ± 2.0	0.686
		TG/GG	2.9 ± 1.6		3.2 ± 1.7		4.4 ± 1.7		5.9 ± 1.8		5.3 ± 2.3		4.6 ± 1.9	
	Recessive	TT/TG	2.9 ± 1.6	0.932	3.1 ± 1.6	0.665	4.4 ± 1.6	0.909	5.8 ± 1.7	0.369	5.0 ± 2.4	0.011	4.6 ± 1.8	0.901
		GG	2.9 ± 1.7		3.2 ± 1.8		4.4 ± 1.9		6.0 ± 1.8		5.7 ± 2.2		4.6 ± 2.1	
*IL10* (rs1878672)	Dominant	GG	2.9 ± 1.8	0.782	3.1 ± 1.6	0.756	4.4 ± 1.6	0.868	5.9 ± 1.8	0.787	5.5 ± 2.4	0.345	4.8 ± 1.9	0.270
		GC/CC	2.8 ± 1.5		3.2 ± 1.7		4.4 ± 1.8		5.9 ± 1.7		5.2 ± 2.3		4.5 ± 1.9	
	Recessive	GG/GC	3.0 ± 1.5	0.068	3.2 ± 1.5	0.316	4.5 ± 1.6	0.417	5.9 ± 1.7	0.969	5.4 ± 2.2	0.906	4.8 ± 1.7	0.035
		CC	2.1 ± 2.1		2.8 ± 2.2		4.1 ± 2.3		5.9 ± 1.9		4.8 ± 2.8		3.6 ± 2.7	
*IL12B* (rs3212227)	Dominant	TT	2.9 ± 1.8	0.620	3.2 ± 1.8	0.614	4.4 ± 1.8	0.872	5.8 ± 1.8	0.360	5.2 ± 2.3	0.470	4.7 ± 2.0	0.698
		TG/GG	2.8 ± 1.4		3.1 ± 1.4		4.4 ± 1.6		6.0 ± 1.6		5.4 ± 2.3		4.6 ± 1.8	
	Recessive	TT/TG	3.1 ± 1.7	0.932	4.4 ± 1.7	0.761	5.9 ± 1.7	0.700	5.3 ± 2.3	0.870	4.7 ± 1.9	0.207	2.9 ± 1.7	0.160
		GG	3.1 ± 1.0		4.3 ± 1.6		6.0 ± 2.0		5.4 ± 2.1		4.0 ± 1.3		2.3 ± 1.0	
*IL17A* (rs2275913)	Dominant	GG	2.7 ± 1.7	0.308	3.1 ± 1.6	0.862	4.5 ± 1.7	0.303	5.7 ± 1.7	0.268	5.3 ± 2.2	0.930	4.5 ± 1.9	0.489
		GA/AA	3.0 ± 1.6		3.2 ± 1.7		4.3 ± 1.8		6.0 ± 1.8		5.3 ± 2.4		4.7 ± 1.9	
	Recessive	GG/GA	2.9 ± 1.6	0.446	3.2 ± 1.6	0.173	4.4 ± 1.7	0.381	5.9 ± 1.8	0.898	5.3 ± 2.3	0.714	4.6 ± 1.9	0.401
		AA	2.6 ± 1.7		2.7 ± 1.9		4.1 ± 1.8		5.9 ± 1.6		5.1 ± 2.3		4.9 ± 2.1	

CTLA-4, cytotoxic T-lymphocyte antigen 4; IL, interleukin; SD, standard deviation; SNP, single-nucleotide polymorphism; TLR, toll-like receptor; TNF, tumor necrosis factor; TTV, torque teno virus.

Next, we analyzed if there was any association between candidate SNPs and the presence of undectectable plasma TTV DNAemia at baseline (before the initiation of immunosuppressive therapy). Seven (3.7%) patients had pre-transplant TTV DNA levels below the LLoD. We observed that carriers of the minor C allele of the *IL10* (rs1878672) SNP in the homozygous state (CC) were more likely to have undetectable baseline TTV DNAemia compared to recipients bearing the reference G allele (GG/GC) [12.5% (3/24) versus 2.5% (4/163), respectively; nominal *p*-value = 0.046]. There were also significant differences within the *TLR3* (rs3775291) SNP, since all the 7 patients with undetectable TTV DNAemia harbored the minor T allele either in the heterozygous or the homozygous state [7.1% (7/99) versus 0.0% (0/88) for CT/TT and CC carriers; nominal *p*-value = 0.015]. Finally, the minor allele of *CD209* (rs4804803) in the homozygous state was also associated with undetectable TTV DNAemia before transplantation [37.5% (5/8) versus 2.2% (4/179) for GG and AA/AG carriers; nominal *p*-value = 0.0017]. Nevertheless, it should be noted that only the latter association was below the Bonferroni-corrected *p*-value threshold for statistical significance (which was settled at 0.00178) (Table 4). We further assessed whether the impact of the *CD209* (rs4804803) SNP remained significant after adjusting for recipient demographics and pre-transplant clinical characteristics also associated with undetectable TTV DNAemia at baseline (Supplementary Table S2). In a logistic regression model that included recipient age and previous renal replacement therapy as covariates, the presence of the minor G allele of *CD209* (rs4804803) in the homozygous state was still significantly associated with pre-transplant TTV DNA levels below the LLoD (adjusted OR: 36.96; 95% CI: 4.72–289.67; *p*-value = 0.001).

**TABLE 4:** Association between undetectable TTV DNAemia at the baseline (pre-transplant) assessment and candidate SNPs in dominant (heterozygous and homozygous) and recessive (homozygous only) models.

SNP (ID number)	Model	Genotype	Undetectable TTV DNAemia at baseline [n (%)]		*p*-value
			No (n = 180)	Yes (n = 7)	
*CTLA4* (rs5742909)	Dominant	CC	149 (82.8)	6 (85.7)	0.840
		CT/TT	31 (17.2)	1 (14.3)	
	Recessive	CC/CT	176 (97.8)	7 (100.0)	0.690
		TT	4 (2.2)	0 (0.0)	
*CTLA4* (rs231775)	Dominant	AA	95 (52.8)	3 (42.9)	0.606
		AG/GG	85 (47.2)	4 (57.1)	
	Recessive	AA/AG	163 (90.6)	7 (100.0)	0.394
		GG	17 (9.4)	0 (0.0)	
*TLR3* (rs3775291)	Dominant	CC	88 (48.9)	0 (0.0)	0.015
		CT/TT	92 (51.1)	7 (100.0)	
	Recessive	CC/CT	145 (80.6)	5 (71.4)	0.304
		TT	26 (19.4)	2 (28.6)	
*TLR9* (rs5743836)	Dominant	AA	130 (72.2)	4 (57.1)	0.385
		AG/GG	50 (27.8)	3 (42.9)	
	Recessive	AA/AG	171 (95)	7 (100.0)	0.544
		GG	9 (5)	0 (0.0)	
*TLR9* (rs352139)	Dominant	TT	47 (26.1)	3 (42.9)	0.326
		TC/CC	133 (73.9)	4 (57.1)	
	Recessive	TT/TC	129 (71.7)	4 (57.1)	0.405
		CC	51 (28.3)	3 (42.9)	
*CD209* (rs735240)	Dominant	GG	48 (26.7)	3 (42.9)	0.345
		GA/AA	132 (73.3)	4 (57.1)	
	Recessive	GG/GA	134 (74.4)	6 (4.3)	0.500
		AA	46 (25.6)	1 (2.1)	
*CD209* (rs4804803)	Dominant	AA	105 (58.3)	3 (42.9)	0.416
		AG/GG	75 (41.7)	4 (57.1)	
	Recessive	AA/AG	175 (97.2)	4 (57.1)	0.0017
		GG	5 (2.8)	3 (42.9)	
*IFNL3* (rs12979860)	Dominant	CC	83 (46.1)	3 (42.9)	0.865
		CT/TT	97 (53.9)	4 (57.1)	
	Recessive	CC/CT	152 (84.4)	7 (4.4)	0.258
		TT	28 (15.6)	0 (0.0)	
*IFNL3* (rs8099917)	Dominant	TT	129 (71.7)	3 (42.9)	0.101
		TG/GG	51 (28.3)	4 (57.1)	
	Recessive	TT/TG	172 (95.6)	7 (100.0)	0.569
		GG	8 (4.4)	0 (0.0)	
*TNF* (rs1800629)	Dominant	GG	137 (76.1)	4 (57.1)	0.253
		GA/AA	43 (23.9)	3 (42.9)	
	Recessive	GG/GA	178 (98.9)	7 (3.8)	0.779
		AA	2 (1.1)	0 (0.0)	
*IL10* (rs1800872)	Dominant	TT	16 (8.9)	2 (28.6)	0.083
		TG/GG	164 (91.1)	5 (71.4)	
	Recessive	TT/TG	109 (60.6)	4 (57.1)	0.856
		GG	71 (39.4)	3 (42.9)	
*IL10* (rs1878672)	Dominant	GG	66 (36.7)	3 (42.9)	0.739
		GC/CC	114 (63.3)	4 (57.1)	
	Recessive	GG/GC	159 (88.3)	4 (57.1)	0.046
		CC	21 (11.7)	3 (42.9)	
*IL12B* (rs3212227)	Dominant	TT	89 (49.4)	4 (57.1)	0.689
		TG/GG	91 (5406)	3 (42.9)	
	Recessive	TT/TG	165 (91.7)	7 (100.0)	0.426
		GG	15 (8.3)	0 (0.0)	
*IL17A* (rs2275913)	Dominant	GG	76 (42.2)	4 (57.1)	0.434
		GA/AA	104 (57.8)	3 (42.9)	
	Recessive	GG/GA	157 (87.2)	5 (71.4)	0.228
		AA	23 (12.8)	2 (28.6)	

CTLA-4, cytotoxic T-lymphocyte antigen 4; IL, interleukin; SNP, single-nucleotide polymorphism; TLR, toll-like receptor; TNF, tumor necrosis factor; TTV, torque teno virus.

In order to better characterize the genetic determinants of post-transplant TTV viral kinetics, we compared peak TTV DNA levels through different time intervals according to candidate SNPs. The only apparent correlation was observed for *TNF* (rs1800629), with carriers of the minor allele either in the heterozygous or homozygous state showing lower peak levels during the first 3 post-transplant months (4.2 ± 1.5 versus 5.0 ± 1.8 log_10_ copies/mL for GA/AA and GG carriers; nominal *p*-value = 0.008) (Supplementary Table S3). This comparison, however, did not attain the Bonferroni-corrected significance level (settled at 0.00059). Accordingly, recipients bearing the minor A allele of this SNP also showed a non-significant trend—by applying the Bonferroni correction—towards a lower AUC for plasma TTV DNAemia through month 6 (5.8 ± 1.7 versus 6.8 ± 1.7 log_10_ copies/mL for GA/AA and GG carriers; nominal *p*-value = 0.007) (Supplementary Table S4). Finally, no significant differences at the Bonferroni-adjusted α level were found in increments (Δ) in TTV DNA levels from baseline to day 15 and months 1, 3, 6 and 12 after transplantation either (Supplementary Table S5).

## Discussion

There is increasing evidence on the usefulness of TTV as a surrogate marker of the immune status in a variety of clinical scenarios (Martin-Lopez et al., 2020; Honorato et al., 2021; Studenic et al., 2021), in particular SOT (Fernandez-Ruiz et al., 2019; Redondo et al., 2022a; Eldar-Yedidia et al., 2022; Jaksch et al., 2022), under the rationale that the T-cell-mediated immunity plays an instrumental role in controlling viral replication. Nevertheless, the relative contribution of the innate system—and its genetic determinants—has not been characterized so far. To our knowledge only three previous works have analyzed the impact of genetic polymorphisms on TTV replication in HSCT recipients (with two studies from the same group) and people living with human immunodeficiency virus (HIV) (Prasetyo et al., 2015; Ramzi et al., 2019; Ramzi et al., 2021). Ramzi et al. (2019); Ramzi et al. (2021) found a correlation between SNPs in *IL10*, *CTLA4* and *TNF* genes and TTV infection in allogeneic HSCT recipients. In detail, the heterozygote genotypes of *IL10* rs1800872 (−592C/A) and *CTLA4* rs231775 (+49 A/G) were associated with a higher prevalence of TTV DNAemia, whereas the A allele of *TNF* rs1800629 (−308G/A) had a protective effect. On the other hand, Prasetyo et al. reported a correlation between the *APOBEC3B* deletion polymorphism status and TTV, HBV and HCV infection among HIV patients (Prasetyo et al., 2015; Ramzi et al., 2019; Ramzi et al., 2021). The present investigation is the first to evaluate to what extent the kinetics of TTV DNA levels following KT are influenced by SNPs in genes coding for PRRs (TLR3, TLR9 and CD209), ILs and cytokines (IL-12B, IL-17, IL-10, TNF), IFN-λ3 (IL-28B) and the costimulatory receptor CTLA-4). These candidate SNPs were chosen on the basis of prior studies performed in the HSCT population (Ramzi et al., 2019; Ramzi et al., 2021) or due to their well-established involvement in other viral infections in SOT recipients (Redondo et al., 2022d).

We found no clear association between any of the SNP genotypes considered and various parameters reflecting viral kinetics after transplantation, such as mean and peak DNA levels or AUCs for plasma TTV DNAemia during discrete periods, or absolute increments from baseline. The significant differences observed for the minor A allele of *TNF* (rs1800629) in terms of lower peak DNA levels and AUC through months 3 and 6 were not consistent across the entire post-transplant monitoring period and did not survive correction for multiple comparisons. Interestingly, Ramzi et al. (2010) Reported that the A allele of the *TNF* (rs1800629) SNP was associated with undetectable TTV DNAemia in a single-center cohort of HSCT recipients (OR: 0.46; 95% CI: 0.22–0.96; *p*-value = 0.025), although the timing for monitoring was unclear and no correction for multiple testing was performed. In line with our results, the same group observed no apparent impact of genotypes of *CTLA4* (rs5742909) on the incidence of TTV infection after HSCT (Ramzi et al., 2019; Ramzi et al., 2021).

In addition to the longitudinal post-transplant monitoring of TTV replication, we have specifically investigated the associations between candidate SNPs and the presence of TTV infection at the baseline assessment, before immunosuppressive therapy was initiated. The cross-sectional comparison at this time point would reveal the potential role of genetic predisposition to TTV among ESRD patients in the absence of iatrogenic immunosuppression. In contrast to the negative results observed for the post-transplant period, we found that the minor G allele of *CD209* (rs4804803) in the homozygous state exerted a protective effect even after the Bonferroni correction, and that this association with undetectable TTV DNAemia at baseline remained significant after adjusting for clinical covariates. Although caution must be exercised due to the low number of patients with pre-transplant TTV DNA levels below the LLoD, this finding is in accordance with a recent study by our group showing a protective effect against BK polyomavirus viremia linked to the G allele of *CD209* (rs4804803) after KT (Redondo et al., 2022c). In addition, the minor G allele has been also associated with a lower susceptibility to tuberculosis (Vannberg et al., 2008) and severe dengue (Sakuntabhai et al., 2005). The *CD209* gene codes for DC-SIGN, a transmembrane PRR belonging to the CLR family. It has been described that the presence of the G allele negatively affects gene transcription, thus downregulating the synthesis of DC-SIGN in dendritic cells (Sakuntabhai et al., 2005). In addition, DC-SIGN acts as the cell receptor for many viruses through its high affinity binding of mannose-containing carbohydrates expressed by viral glycoproteins (Lin et al., 2003). In view of the non-enveloped structure of anelloviruses, the mechanistic explanation for the association found between the *CD209* (rs4804803) SNP and baseline TTV DNAemia remains to be determined and demands further investigation in healthy subjects (e.g., blood donors).

Despite its large sample size, frequent TTV DNA monitoring and comprehensive set of SNPs screened, some limitations to our study should be acknowledged. As previously described (Redondo et al., 2022a; Jaksch et al., 2022), the vast majority of recipients had TTV replication early after transplantation. Thus, associations between genetic polymorphisms and undetectable TTV DNAemia (below the LLoD of the PCR assay) were only analyzed at baseline. Since the relatively high number of SNPs imposed stringent thresholds for statistical significance, false-negative results due to insufficient statistical power cannot be excluded, particularly for those SNPs—such as *CTLA4* (rs5742909) or *TNF* (rs1800629)—with very low absolute numbers of patients bearing the corresponding minor alleles.

In conclusion, the G allele of *CD209* (rs4804803) in the homozygous state would play a protective role against TTV in non-immunocompromised patients listed for KT, whereas no significant associations have been found during the post-transplant period for any of the studied SNPs. Thus, the present results support the conception that variations in plasma TTV DNA levels after KT are mainly driven by the effect of immunosuppressive therapy rather than by underlying genetic predisposition, reinforcing its clinical usefulness as a surrogate marker of immunosuppression. Post-transplant TTV replication kinetics seems to be mainly under the control of the adaptive immune responses, with no meaningful effect of SNPs in genes orchestrating innate arm.

## Data Availability

The data that support the findings of this study are available from the corresponding author upon reasonable request. SNP genotyping data are registered in the BioProject database under the ID PRJNA898147.

## Abbreviations

AUC, area the under curve; BKPyV, BK polyomavirus; CI, confidence interval; CpG, cytosine-phosphate-guanine; CMV, cytomegalovirus; D, donor; dsRNA, double-stranded RNA; ESRD, end-stage renal disease; HBV, hepatitis B virus; HCV, hepatitis C virus; HIV, human immunodeficiency virus; HSCT, hematopoietic stem cell transplantation; IQR, interquartile range; KT, kidney transplantation; LLoD, lower limit of detection; OR, odds ratio; PAMP, pathogen-associated molecular pattern; PCR, polymerase chain reaction; R, recipient; PRR, pattern recognition receptor; SD, standard deviation; SNP, single-nucleotide polymorphism; SOT, solid organ transplantation; TLR, toll-like receptor; TTV, Torque teno virus.
