# Supplementary material for: Impact of polymorphisms in genes orchestrating innate immune responses on replication kinetics of Torque teno virus after kidney transplantation
Source: Front Genet. 2022 Nov 22;13:1069890. doi: 10.3389/fgene.2022.1069890 (PMC9723237; doi:10.3389/fgene.2022.1069890)
Supplement: Supplementary file 1 [file DataSheet1.PDF]

<u>Supporting Material</u>

Supplementary Methods

Immunosuppression and prophylaxis regimens

All kidney transplant recipients of organs from donors after circulatory death underwent induction therapy with intravenous (IV) rabbit antithymocyte globulin (ATG-Fresenius, 1.25 mg/Kg daily for 5-7 days), with delayed introduction of a calcineurin inhibitor (CNI) from post-transplant day 6. Recipients at high immunological risk also received ATG induction for 1-3 days with early CNI initiation from post-transplant day 0. Basiliximab (20 mg on days 0 and 4) with delayed CNI introduction from post-transplant day 5 was reserved to patients at high risk for CNI-related nephrotoxicity (i.e. older age or pre-transplant comorbidities). Maintenance immunosuppression consisted of tacrolimus (0.1 mg/Kg daily, adjusted to a target trough level of 10-15 ng/mL during the first month and 5-10 ng/mL thereafter); mycophenolic acid (360 mg twice daily); and prednisone (1 mg/Kg daily with progressive tapering). Conversion to mammalian target of rapamycin (mTOR) inhibitor-based regimens with reduced-dose tacrolimus (target trough level of 3-6 ng/mL) was performed on an individual basis for recipients experiencing severe CNI-related adverse effects, difficult-to-treat cytomegalovirus (CMV) infection or post-transplant cancer.

All patients received preoperatively a single dose of IV cefazolin (which was replaced with ciprofloxacin in those with ß-lactam hypersensitivity). Prophylaxis for *Pneumocystis jirovecii* pneumonia with trimethoprim-sulfamethoxazole (160/800 mg three times weekly) or monthly pentamidine was given for 9 months. In patients at high-risk for CMV disease (serology mismatch [donor positive (D+) and recipient negative (R-)] or induction therapy with ATG), either IV ganciclovir (5 mg/Kg daily) or oral valganciclovir (900 mg daily) were administered as universal prophylaxis for 3 to 6 months. Intermediate-risk patients (R+ without T-cell-depleting therapy) were monitored every 2-4 weeks for CMV infection with a PCR-based assay (RealStar® CMV PCR kit, Altona Diagnostics, Hamburg, Germany), and preemptive therapy with IV ganciclovir (5

mg/Kg/12 hours) or oral valganciclovir (900 mg/12 hours) was administered for at least 2 weeks in presence of high-level (>1.000 IU/mL) or rapidly increasing viral load. (Val)ganciclovir dose was adjusted according to renal function when necessary [1,2].

References

1. Kotton CN, Kumar D, Caliendo AM, Asberg A, Chou S, Danziger-Isakov L, et al. Updated international consensus guidelines on the management of cytomegalovirus in solid-organ transplantation. Transplantation 2013; 96: 333-360.
2. Torre-Cisneros J, Aguado JM, Castón JJ, Almenar L, Alonso A, Cantisán S, et al. Management of cytomegalovirus infection in solid organ transplant recipients: SET/GESITRA-SEIMC/REIPI recommendations. Transplant Rev (Orlando) 2016; 30: 119-143.

Supplementary Results

Table S1. Genotypic frequencies of the candidate SNPs.

Gene (SNP database ID number)	Genotype	N	(%)
CTL4 (rs5742909)	CC	180	81
	CT	36	16
	TT	5	2
CTL4 (rs231775)	AA	112	51
	AG	88	40
	GG	21	10
TLR3 (rs3775291)	CC	108	49
	CT	84	38
	TT	29	13
TLR9 (rs5743836)	AA	160	72
	AG	51	23
	GG	10	5
TLR9 (rs352139)	TT	64	29
	TC	96	43
	CC	61	28
CD209 (rs735240)	GG	61	28
	GA	108	49
	AA	52	24
CD209 (rs4804803)	AA	129	58
	AG	80	36
	GG	12	5
IL28B (rs12979860)	CC	106	48
	CT	86	39
	TT	32	14
IL28B (rs8099917)	TT	160	72
	TG	52	24
	GG	9	4
TNF (rs1800629)	GG	172	78
	GA	46	21
	AA	3	1
IL10 (rs1800872)	TT	22	10
	TG	108	49
	GG	91	41
IL10 (rs1878672)	GG	83	38
	GC	107	48
	CC	31	14

IL12B (rs3212227)	TT	110	50
	TG	93	42
	GG	18	8
IL17A (rs2275913)	GG	103	47
	GA	90	41
	AA	28	13

CTLA-4: cytotoxic T-lymphocyte antigen 4; IL: interleukin; SNP: single-nucleotide polymorphism; TLR: toll-like receptor; TNF: tumor necrosis factor.

Table S2. Comparison of demographics and clinical characteristics between evaluable recipients with or without undetectable TTV DNAemia at the baseline (pre-transplant) assessment (n = 187).

**Variable**	**Undetectable TTV DNAemia at baseline**		*P*-value
	No (n = 180)	Yes (n = 7)	
Age of recipient, years [mean ± SD]	54.4 ± 15.9	45.7 ± 6.6	0.012
Male gender of recipient [n (%)]	128 (71.1)	4 (57.1)	0.421
Current or prior smoking history [n (%)]	74 (41.1)	3 (42.9)	1.000
Pre-transplant chronic co-morbidities [n (%)]			
Hypertension	152 (84.9)	7 (100.0)	0.596
Diabetes mellitus	58 (32.2)	1 (14.3)	0.435
Chronic lung disease	26 (14.4)	0 (0.0)	0.596
Coronary heart disease	20 (11.1)	1 (14.3)	0.572
Other chronic heart disease	36 (20.0)	0 (0.0)	0.349
Peripheral arterial disease	15 (8.3)	1 (14.3)	0.471
Cerebrovascular disease	12 (6.7)	0 (0.0)	1.000
Previous solid organ transplantation [n (%)]	20 (11.2)	2 (28.6)	0.914
Underlying end-stage renal disease [n (%)]			
Glomerulonephritis	41 (22.8)	2 (28.6)	0.662
Diabetic nephropathy	36 (20.0)	1 (14.3)	1.000
Polycystic kidney disease	22 (12.2)	1 (14.3)	1.000
Chronic interstitial nephropathy	9 (5.0)	0 (0.0)	1.000
Unknown	22 (12.2)	1 (14.3)	1.000
Other	15 (8.3)	1 (14.3)	0.471
Positive CMV serostatus [n (%)]	152 (84.4)	5 (71.4)	0.312
Positive HCV serostatus [n (%)]	15 (8.3)	1 (14.3)	0.471
Positive HIV serostatus [n (%)]	2 (1.1)	0 (0.0)	1.000
Pre-transplant renal replacement therapy [n (%)]	158 (87.8)	4 (57.1)	0.052
Hemodialysis	132 (83.5)	4 (100.0)	1.000
Continuous ambulatory peritoneal dialysis	26 (16.5)	0 (0.0)	
Time on dialysis, months [median (IQR)]	17.6 (9.3 – 34.4)	43.7 (10.8 – 78.9)	0.356

CMV: cytomegalovirus; HCV: hepatitis C virus; HIV: human immunodeficiency virus; IQR: interquartile range; SD: standard deviation; TTV: torque teno virus.

Table S3. Areas under the curve for plasma TTV DNA through different post-transplant periods according to candidate SNPs.

SNP (ID number)	Model	Genotype	AUC for plasma TTV DNA, log_{10} copies/mL (mean ± SD)					
			First month	*P*-value	First 3 months	*P*-value	First 6 months	*P*-value
CTLA4 (rs5742909)	Dominant	CC<br>CT/TT	4.9 ± 1.7<br>4.6 ± 1.6	0.303	6.7 ± 1.8<br>6.2 ± 1.8	0.269	7.9 ± 1.6<br>7.7 ± 1.7	0.448
	Recessive	CC/CT<br>TT	4.8 ± 1.7<br>4.9 ± 1.7	0.906	6.6 ± 1.8<br>6.2 ± 1.6	0.685	7.8 ± 1.6<br>9.0 ± 1.2	0.125
CTLA4 (rs231775)	Dominant	AA<br>AG/GG	4.8 ± 1.6<br>4.9 ± 1.8	0.612	6.6 ± 1.8<br>6.5 ± 1.8	0.628	7.8 ± 1.5<br>7.9 ± 1.7	0.755
	Recessive	AA/AG<br>GG	4.8 ± 1.7<br>5.1 ± 1.6	0.516	6.6 ± 1.7<br>5.9 ± 2.2	0.168	7.9 ± 1.6<br>8.0 ± 2.0	0.726
TLR3 (rs3775291)	Dominant	CC<br>CT/TT	4.8 ± 1.5<br>4.8 ± 1.8	0.955	6.5 ± 2.0<br>6.6 ± 1.6	0.784	8.0 ± 1.8<br>7.8 ± 1.5	0.379
	Recessive	CC/CT<br>TT	4.8 ± 1.5<br>4.8 ± 2.5	0.858	6.6 ± 1.8<br>6.3 ± 1.9	0.448	7.9 ± 1.7<br>7.8 ± 1.5	0.733
TLR9 (rs5743836)	Dominant	AA<br>AG/GG	4.8 ± 1.7<br>4.7 ± 1.6	0.517	6.5 ± 1.8<br>7.0 ± 1.7	0.164	7.8 ± 1.7<br>8.0 ± 1.4	0.521
	Recessive	AA/AG<br>GG	4.8 ± 1.7<br>4.5 ± 1.4	0.545	6.6 ± 1.8<br>6.5 ± 1.2	0.896	7.9 ± 1.6<br>7.6 ± 1.8	0.554
TLR9 (rs352139)	Dominant	TT<br>TC/CC	5.1 ± 1.9<br>4.7 ± 1.6	0.164	7.1 ± 2.3<br>6.4 ± 1.5	0.030	7.9 ± 1.8<br>7.9 ± 1.6	0.944
	Recessive	TT/TC<br>CC	4.9 ± 1.7<br>4.7 ± 1.6	0.468	6.6 ± 1.9<br>6.5 ± 1.5	0.835	7.9 ± 1.7<br>7.8 ± 1.4	0.850
CD209 (rs735240)	Dominant	GG	4.7 ± 2.0	0.472	6.6 ± 1.9	0.935	7.7 ± 1.7	0.498

		GA/AA	4.9 ± 1.6		6.6 ± 1.7		7.9 ± 1.6	
	Recessive	GG/GA<br>AA	4.7 ± 1.7<br>5.3 ± 1.4	0.034	6.5 ± 1.7<br>7.0 ± 2.0	0.149	7.8 ± 1.7<br>7.9 ± 1.4	0.748
CD209 (rs4804803)	Dominant	AA<br>AG/GG	4.9 ± 1.6<br>4.8 ± 1.8	0.825	6.6 ± 1.8<br>6.5 ± 1.8	0.657	7.9 ± 1.5<br>7.8 ± 1.7	0.825
	Recessive	AA/AG<br>GG	4.9 ± 1.6<br>4.1 ± 2.8	0.331	6.6 ± 1.8<br>7.0 ± 2.3	0.503	7.9 ± 1.6<br>8.0 ± 2.0	0.758
IFNL3 (rs12979860)	Dominant	CC<br>CT/TT	5.0 ± 1.6<br>4.6 ± 1.7	0.093	6.8 ± 1.7<br>6.4 ± 1.9	0.197	8.0 ± 1.6<br>7.7 ± 1.6	0.145
	Recessive	CC/CT<br>TT	4.8 ± 1.7<br>4.8 ± 1.6	0.939	6.7 ± 1.8<br>6.1 ± 1.7	0.249	7.9 ± 1.6<br>7.9 ± 1.6	0.981
IFNL3 (rs8099917)	Dominant	TT<br>TG/GG	4.9 ± 1.6<br>4.6 ± 1.9	0.181	6.7 ± 1.8<br>6.3 ± 1.8	0.205	7.9 ± 1.7<br>7.7 ± 1.5	0.331
	Recessive	TT/TG<br>GG	4.8 ± 1.7<br>5.1 ± 1.3	0.616	6.6 ± 1.8<br>7.2 ± 2.1	0.449	7.9 ± 1.6<br>8.2 ± 1.1	0.639
TNF (rs1800629)	Dominant	GG<br>GA/AA	5.0 ± 1.6<br>4.4 ± 1.8	0.061	6.8 ± 1.7<br>5.8 ± 1.7	0.007	7.9 ± 1.6<br>7.8 ± 1.7	0.711
	Recessive	GG/GA<br>AA	4.8 ± 1.7<br>5.4 ± 1.8	0.574	6.6 ± 1.8<br>5.7 ± 1.3	0.617	7.9 ± 1.6<br>8.3 ± 1.6	0.666
IL10 (rs1800872)	Dominant	TT<br>TG/GG	4.7 ± 1.4<br>4.8 ± 1.7	0.766	7.0 ± 1.4<br>6.5 ± 1.8	0.417	7.9 ± 1.4<br>7.9 ± 1.7	0.813
	Recessive	TT/TG<br>GG	4.8 ± 1.6<br>4.9 ± 1.8	0.808	6.6 ± 1.8<br>6.6 ± 1.7	0.937	7.8 ± 1.6<br>8.0 ± 1.7	0.291
IL10 (rs1878672)	Dominant	GG<br>GC/CC	4.8 ± 1.7<br>4.9 ± 1.7	0.822	6.6 ± 2.0<br>6.6 ± 1.6	0.884	7.8 ± 1.8<br>7.9 ± 1.5	0.907
	Recessive	GG/GC	4.8 ± 1.6	0.705	6.6 ± 1.8	0.564	7.9 ± 1.6	0.900

		CC	4.7 ± 2.0		6.3 ± 1.7		7.8 ± 1.8	
IL12B (rs3212227)	Dominant	TT<br>TG/GG	4.9 ± 1.8<br>4.7 ± 1.6	0.403	6.6 ± 2.0<br>6.6 ± 1.6	0.959	7.7 ± 1.7<br>8.0 ± 1.6	0.098
	Recessive	TT/TG<br>GG	4.8 ± 1.7<br>4.7 ± 1.3	0.665	6.5 ± 1.8<br>7.1 ± 1.4	0.322	7.9 ± 1.6<br>8.0 ± 1.9	0.671
IL17A (rs2275913)	Dominant	GG<br>GA/AA	4.9 ± 1.6<br>4.8 ± 1.8	0.586	6.8 ± 1.6<br>6.4 ± 1.9	0.163	7.9 ± 1.5<br>7.9 ± 1.7	0.946
	Recessive	GG/GA<br>AA	4.9 ± 1.6<br>4.6 ± 1.9	0.404	6.6 ± 1.7<br>6.1 ± 2.0	0.271	7.9 ± 1.7<br>7.8 ± 1.4	0.831

AUC: area under the curve; CTLA-4: cytotoxic T-lymphocyte antigen 4; IL: interleukin; SNP: single-nucleotide polymorphism; TLR: toll-like receptor; TNF: tumor necrosis factor; TTV: torque teno virus.

Table S4. Increments (Δ) in TTV DNA levels from baseline to different points after transplantation according to candidate SNPs.

**SNP (ID number)**	**Model**	**Genotype**	**Increment (Δ) in TTV DNA levels, $\log_{10}$ copies/mL (mean ± SD)**									
			**Baseline → day 15**	*P*-value	**Baseline → month 1**	*P*-value	**Baseline → moth 3**	*P*-value	**Baseline → moth 6**	*P*-value	**Baseline → moth 12**	*P*-value
CTLA4 (rs5742909)	Dominant	CC<br>CT/TT	2.4 ± 0.9<br>2.7 ± 1.5	0.344	3.5 ± 1.2<br>3.2 ± 0.7	0.487	4.5 ± 1.8<br>4.5 ± 2.1	0.894	4.9 ± 2.1<br>5.0 ± 1.4	0.921	4.3 ± 1.9<br>4.6 ± 1.8	0.537
	Recessive	CC/CT<br>TT	2.5 ± 1.1<br>2.0	0.626	3.5 ± 1.1<br>2.9 ± 0.8	0.516	4.5 ± 1.8<br>4.2 ± 2.6	0.850	4.9 ± 2.0<br>5.1 ± 1.7	0.873	4.4 ± 1.9<br>4.3 ± 2.0	0.971
CTLA4 (rs231775)	Dominant	AA<br>AG/GG	2.5 ± 1.1<br>2.5 ± 1.0	0.863	3.5 ± 1.1<br>3.4 ± 1.2	0.819	4.4 ± 1.8<br>4.5 ± 1.8	0.700	4.9 ± 1.8<br>5.0 ± 2.1	0.922	4.4 ± 1.7<br>4.4 ± 2.0	0.936
	Recessive	AA/AG<br>GG	2.5 ± 1.1<br>2.4 ± 0.5	0.802	3.5 ± 1.2<br>3.3 ± 0.8	0.708	4.5 ± 1.8<br>3.8 ± 1.3	0.270	4.9 ± 1.9<br>5.4 ± 2.2	0.428	4.4 ± 1.9<br>4.4 ± 1.8	0.975
TLR3 (rs3775291)	Dominant	CC<br>CT/TT	2.4 ± 1.1<br>2.6 ± 1.0	0.333	3.3 ± 1.1<br>3.5 ± 1.2	0.473	4.6 ± 1.7<br>4.3 ± 1.9	0.442	5.2 ± 2.1<br>4.7 ± 1.8	0.094	4.5 ± 2.0<br>4.2 ± 1.8	0.302
	Recessive	CC/CT<br>TT	2.5 ± 1.1<br>2.4 ± 1.0	0.781	3.5 ± 1.1<br>3.3 ± 1.4	0.666	4.6 ± 1.9<br>3.8 ± 1.3	0.118	5.0 ± 1.9<br>4.4 ± 2.2	0.200	4.4 ± 1.8<br>4.1 ± 2.0	0.543
TLR9 (rs5743836)	Dominant	AA<br>AG/GG	2.4 ± 1.0<br>2.7 ± 1.2	0.257	3.6 ± 1.1<br>3.1 ± 1.1	0.084	4.2 ± 1.6<br>5.2 ± 2.3	0.058	4.8 ± 1.9<br>5.2 ± 2.1	0.257	4.3 ± 1.9<br>4.6 ± 1.9	0.317
	Recessive	AA/AG<br>GG	2.5 ± 1.0<br>2.8 ± 1.5	0.465	3.5 ± 1.1<br>2.6 ± 1.1	0.131	4.5 ± 1.8<br>3.8 ± 1.3	0.390	5.0 ± 2.0<br>4.8 ± 1.8	0.853	4.4 ± 1.9<br>3.8 ± 1.9	0.398
TLR9 (rs352139)	Dominant	TT<br>TC/CC	2.5 ± 0.8<br>2.5 ± 1.2	0.849	3.7 ± 0.9<br>3.3 ± 1.2	0.151	4.7 ± 1.9<br>4.4 ± 1.8	0.366	4.7 ± 1.8<br>5.0 ± 2.0	0.326	4.3 ± 2.1<br>4.4 ± 1.8	0.639
	Recessive	TT/TC<br>CC	2.4 ± 0.8<br>2.8 ± 1.6	0.256	3.5 ± 1.1<br>3.3 ± 1.4	0.513	4.5 ± 1.7<br>4.4 ± 2.0	0.832	5.0 ± 1.9<br>4.7 ± 2.1	0.435	4.3 ± 1.8<br>4.7 ± 1.9	0.241

CD209 (rs735240)	Dominant	GG<br>GA/AA	2.4 ± 1.0<br>2.5 ± 1.1	0.605	3.6 ± 1.4<br>3.4 ± 1.1	0.475	4.5 ± 2.0<br>4.5 ± 1.7	0.996	5.0 ± 2.1<br>4.9 ± 1.9	0.849	4.5 ± 2.1<br>4.4 ± 1.8	0.759
	Recessive	GG/GA<br>AA	2.4 ± 1.0<br>2.7 ± 1.4	0.406	3.4 ± 1.1<br>3.5 ± 1.1	0.731	4.4 ± 1.8<br>4.5 ± 1.9	0.840	5.0 ± 2.1<br>4.9 ± 1.6	0.794	4.4 ± 1.9<br>4.3 ± 1.9	0.696
CD209 (rs4804803)	Dominant	AA<br>AG/GG	2.6 ± 1.1<br>2.4 ± 1.0	0.347	3.4 ± 1.1<br>3.4 ± 1.2	0.976	4.5 ± 1.9<br>4.4 ± 1.7	0.628	4.8 ± 1.6<br>5.2 ± 2.4	0.264	4.1 ± 1.6<br>4.8 ± 2.1	0.030
	Recessive	AA/AG<br>GG	2.5 ± 1.0<br>4.7 ± 1.0	0.003	3.4 ± 1.1<br>-	NA	4.4 ± 1.8<br>5.0 ± 2.5	0.386	4.9 ± 1.9<br>5.4 ± 3.1	0.533	4.3 ± 1.8<br>5.7 ± 4.0	0.535
IFNL3 (rs12979860)	Dominant	CC<br>CT/TT	2.5 ± 0.9<br>2.5 ± 1.2	0.709	3.5 ± 1.1<br>3.4 ± 1.2	0.851	4.6 ± 1.8<br>4.3 ± 1.8	0.456	4.9 ± 1.9<br>4.9 ± 2.0	0.979	4.3 ± 1.7<br>4.5 ± 2.0	0.510
	Recessive	CC/CT<br>TT	2.6 ± 1.1<br>2.2 ± 0.7	0.207	3.5 ± 1.2<br>3.3 ± 0.9	0.602	4.6 ± 1.9<br>3.6 ± 1.0	0.004	5.0 ± 2.0<br>4.8 ± 1.9	0.610	4.4 ± 1.8<br>4.4 ± 2.2	0.923
IFNL3 (rs8099917)	Dominant	TT<br>TG/GG	2.4 ± 1.0<br>2.6 ± 1.2	0.393	3.5 ± 1.1<br>3.2 ± 1.3	0.237	4.6 ± 1.8<br>4.2 ± 1.8	0.294	5.0 ± 2.0<br>4.8 ± 1.9	0.463	4.4 ± 1.9<br>4.2 ± 1.7	0.560
	Recessive	TT/TG<br>GG	2.5 ± 1.1<br>3.0 ± 0.3	0.364	3.4 ± 1.1<br>3.4 ± 1.3	0.881	4.4 ± 1.8<br>5.7 ± 2.8	0.230	5.0 ± 2.0<br>4.6 ± 1.3	0.625	4.4 ± 1.9<br>3.8 ± 1.1	0.515
TNF (rs1800629)	Dominant	GG<br>GA/AA	2.5 ± 1.1<br>2.3 ± 0.9	0.427	3.5 ± 1.1<br>3.4 ± 1.1	0.782	4.6 ± 1.7<br>4.2 ± 2.1	0.379	4.8 ± 1.9<br>5.3 ± 2.1	0.259	4.3 ± 1.7<br>4.6 ± 2.3	0.363
	Recessive	GG/GA<br>AA	2.5 ± 1.1<br>0	NA	3.5 ± 1.1<br>2.3	0.329	4.5 ± 1.8<br>4.2	0.862	5.0 ± 2.0<br>4.3 ± 0.8	0.666	4.4 ± 1.9<br>2.4 ± 1.2	0.132
IL10 (rs1800872)	Dominant	TT<br>TG/GG	2.9 ± 1.3<br>2.5 ± 1.0	0.168	3.2 ± 1.0<br>3.5 ± 1.1	0.456	4.9 ± 3.6<br>4.4 ± 1.6	0.705	4.6 ± 1.6<br>5.0 ± 2.0	0.433	4.8 ± 1.8<br>4.3 ± 1.9	0.384
	Recessive	TT/TG<br>GG	2.6 ± 1.1<br>2.3 ± 1.1	0.272	3.5 ± 1.2<br>3.4 ± 1.1	0.706	4.6 ± 2.0<br>4.3 ± 1.5	0.368	4.7 ± 1.7<br>5.3 ± 2.2	0.076	4.1 ± 1.5<br>4.7 ± 2.2	0.056
IL10 (rs1878672)	Dominant	GG<br>GC/CC	2.6 ± 1.0<br>2.4 ± 1.1	0.372	3.2 ± 1.0<br>3.5 ± 1.2	0.290	5.0 ± 2.2<br>4.2 ± 1.5	0.034	5.1 ± 2.3<br>4.9 ± 1.8	0.523	4.4 ± 2.1<br>4.3 ± 1.7	0.799

	Recessive	GG/GC<br>CC	2.5 ± 0.9<br>2.5 ± 1.6	0.992	3.3 ± 1.0<br>4.1 ± 1.4	0.018	4.4 ± 1.8<br>4.9 ± 2.0	0.481	4.8 ± 2.0<br>5.5 ± 1.9	0.164	4.3 ± 1.8<br>5.0 ± 2.1	0.082
IL12B (rs3212227)	Dominant	TT<br>TG/GG	2.6 ± 1.0<br>2.4 ± 1.1	0.459	3.7 ± 1.1<br>3.2 ± 1.1	0.105	4.6 ± 1.9<br>4.4 ± 1.7	0.549	4.6 ± 1.9<br>5.3 ± 1.9	0.030	4.5 ± 1.9<br>4.3 ± 1.9	0.548
	Recessive	TT/TG<br>GG	2.5 ± 1.1<br>2.4 ± 0.8	0.830	3.3 ± 1.1<br>4.4 ± 0.9	0.036	4.4 ± 1.8<br>5.2 ± 1.7	0.188	4.9 ± 1.9<br>6.0 ± 2.0	0.096	4.3 ± 1.8<br>5.0 ± 2.3	0.161
IL17A (rs2275913)	Dominant	GG<br>GA/AA	2.5 ± 0.9<br>2.5 ± 1.2	0.781	3.6 ± 1.3<br>3.3 ± 1.0	0.200	4.5 ± 1.8<br>4.4 ± 1.8	0.708	4.8 ± 2.0<br>5.0 ± 1.9	0.514	4.4 ± 1.8<br>4.3 ± 1.9	0.842
	Recessive	GG/GA<br>AA	2.4 ± 1.0<br>3.0 ± 1.1	0.078	3.4 ± 1.1<br>3.6 ± 1.0	0.704	4.4 ± 1.7<br>4.8 ± 2.4	0.460	4.9 ± 1.8<br>5.5 ± 2.8	0.383	4.4 ± 1.8<br>4.4 ± 2.0	0.876

CTLA-4: cytotoxic T-lymphocyte antigen 4; IL: interleukin; NA: not applicable; SNP: single-nucleotide polymorphism; TLR: toll-like receptor; TNF: tumor necrosis factor; TTV: torque teno virus.

Table S5. Peak plasma TTV DNA levels at different intervals after transplantation according to candidate SNPs.

SNP (ID number)	Model	Genotype	Peak plasma TTV DNA level, $\log_{10}$ copies/mL (mean ± SD)					
			**First month**	***P*-value**	**First 3 months**	***P*-value**	**First 6 months**	***P*-value**
CTLA4 (rs5742909)	Dominant	CC<br>CT/TT	3.8 ± 1.7<br>3.4 ± 1.9	0.155	4.9 ± 1.8<br>4.6 ± 1.7	0.270	6.0 ± 1.7<br>5.6 ± 1.7	0.143
	Recessive	CC/CT<br>TT	3.7 ± 1.7<br>4.0 ± 1.4	0.686	4.8 ± 1.8<br>5.0 ± 1.2	0.843	5.9 ± 1.7<br>7.3 ± 1.3	0.077
CTLA4 (rs231775)	Dominant	AA<br>AG/GG	3.8 ± 1.6<br>3.7 ± 1.9	0.682	4.9 ± 1.7<br>4.8 ± 1.8	0.667	5.9 ± 1.7<br>5.9 ± 1.8	0.879
	Recessive	AA/AG<br>GG	3.7 ± 1.8<br>4.0 ± 1.4	0.483	4.9 ± 1.7<br>4.5 ± 2.2	0.335	5.9 ± 1.7<br>6.1 ± 2.1	0.691
TLR3 (rs3775291)	Dominant	CC<br>CT/TT	3.7 ± 1.7<br>3.8 ± 1.7	0.576	4.8 ± 1.8<br>4.9 ± 1.7	0.877	6.1 ± 1.9<br>5.7 ± 1.5	0.093
	Recessive	CC/CT<br>TT	3.7 ± 1.6<br>3.7 ± 2.4	0.863	4.9 ± 1.7<br>4.6 ± 2.2	0.429	6.0 ± 1.8<br>5.7 ± 1.5	0.413
TLR9 (rs5743836)	Dominant	AA<br>AG/GG	3.8 ± 1.7<br>3.5 ± 1.8	0.308	4.9 ± 1.8<br>4.7 ± 1.7	0.640	5.8 ± 1.8<br>6.1 ± 1.6	0.264
	Recessive	AA/AG<br>GG	3.7 ± 1.7<br>3.5 ± 1.4	0.675	4.9 ± 1.8<br>4.1 ± 1.3	0.200	6.0 ± 1.7<br>5.3 ± 1.5	0.215
TLR9 (rs352139)	Dominant	TT<br>TC/CC	3.9 ± 2.1<br>3.7 ± 1.5	0.547	5.2 ± 2.3<br>4.7 ± 1.5	0.101	6.1 ± 1.9<br>5.9 ± 1.7	0.516
	Recessive	TT/TC<br>CC	3.7 ± 1.8<br>3.7 ± 1.5	0.774	4.8 ± 1.9<br>4.8 ± 1.5	0.943	5.6 ± 1.9<br>5.9 ± 1.4	0.650

CD209 (rs735240)	Dominant	GG<br>GA/AA	3.7 ± 2.0<br>3.7 ± 1.6	0.833	5.0 ± 1.7<br>4.8 ± 1.8	0.299	5.8 ± 1.7<br>6.0 ± 1.8	0.578
	Recessive	GG/GA<br>AA	3.6 ± 1.8<br>4.0 ± 1.5	0.206	4.8 ± 1.8<br>5.1 ± 1.8	0.271	5.9 ± 1.7<br>5.9 ± 1.8	0.935
CD209 (rs4804803)	Dominant	AA<br>AG/GG	3.8 ± 1.6<br>6.7 ± 1.9	0.599	4.9 ± 1.8<br>4.8 ± 1.7	0.886	5.9 ± 1.7<br>6.0 ± 1.8	0.673
	Recessive	AA/AG<br>GG	3.8 ± 1.6<br>3.0 ± 2.8	0.366	4.8 ± 1.8<br>5.5 ± 1.9	0.211	5.9 ± 1.7<br>5.9 ± 2.1	0.993
IFNL3 (rs12979860)	Dominant	CC<br>CT/TT	3.9 ± 1.6<br>3.6 ± 1.8	0.128	5.1 ± 1.6<br>4.6 ± 1.9	0.044	6.1 ± 1.7<br>5.8 ± 1.8	0.186
	Recessive	CC/CT<br>TT	3.8 ± 1.7<br>3.6 ± 1.8	0.633	4.9 ± 1.8<br>4.4 ± 1.7	0.134	5.9 ± 1.7<br>6.0 ± 1.8	0.773
IFNL3 (rs8099917)	Dominant	TT<br>TG/GG	3.8 ± 1.7<br>3.6 ± 1.8	0.379	4.9 ± 1.8<br>4.6 ± 1.7	0.183	6.0 ± 1.8<br>5.7 ± 1.7	0.237
	Recessive	TT/TG<br>GG	3.7 ± 1.7<br>4.0 ± 1.3	0.582	4.8 ± 1.8<br>5.3 ± 1.7	0.380	5.9 ± 1.8<br>6.6 ± 1.2	0.235
TNF (rs1800629)	Dominant	GG<br>GA/AA	3.8 ± 1.7<br>3.4 ± 1.6	0.111	5.0 ± 1.8<br>4.2 ± 1.5	0.008	6.0 ± 1.7<br>5.8 ± 1.8	0.590
	Recessive	GG/GA<br>AA	3.7 ± 1.7<br>4.2 ± 1.9	0.611	4.8 ± 1.8<br>5.1 ± 0.4	0.799	5.9 ± 1.7<br>6.5 ± 1.6	0.574
IL10 (rs1800872)	Dominant	TT<br>TG/GG	3.5 ± 1.4<br>3.8 ± 1.8	0.529	4.7 ± 1.5<br>4.9 ± 1.8	0.637	5.8 ± 1.3<br>5.9 ± 1.8	0.702
	Recessive	TT/TG<br>GG	3.7 ± 1.7<br>3.8 ± 1.7	0.656	4.9 ± 1.7<br>4.7 ± 1.8	0.442	5.8 ± 1.8<br>6.2 ± 1.7	0.112
IL10 (rs1878672)	Dominant	GG<br>GC/CC	3.6 ± 1.7<br>3.8 ± 1.7	0.506	4.9 ± 1.8<br>4.8 ± 1.8	0.814	5.9 ± 1.9<br>5.9 ± 1.6	0.926

	Recessive	GG/GC<br>CC	3.7 ± 1.7<br>3.7 ± 2.0	0.992	4.9 ± 1.8<br>4.5 ± 1.7	0.325	5.9 ± 1.7<br>5.9 ± 1.9	0.790
IL12B (rs3212227)	Dominant	TT<br>TG/GG	3.8 ± 1.8<br>3.7 ± 1.6	0.724	4.9 ± 1.9<br>4.8 ± 1.6	0.554	5.8 ± 1.8<br>6.1 ± 1.7	0.192
	Recessive	TT/TG<br>GG	3.7 ± 1.8<br>3.7 ± 1.4	0.926	4.8 ± 1.8<br>4.8 ± 1.9	0.838	5.9 ± 1.7<br>6.0 ± 1.9	0.841
IL17A (rs2275913)	Dominant	GG<br>GA/AA	3.8 ± 1.6<br>3.7 ± 1.8	0.489	5.0 ± 1.7<br>4.7 ± 1.8	0.367	5.9 ± 1.5<br>5.9 ± 1.9	0.951
	Recessive	GG/GA<br>AA	3.8 ± 1.7<br>3.3 ± 2.1	0.156	4.9 ± 1.8<br>4.6 ± 1.7	0.481	5.9 ± 1.7<br>6.0 ± 2.2	0.914

CTLA-4: cytotoxic T-lymphocyte antigen 4; IL: interleukin; SNP: single-nucleotide polymorphism; TLR: toll-like receptor; TNF: tumor necrosis factor; TTV: torque teno virus.
